# Autistic Traits Predict Social-Contact Uncertainty in University Students

**DOI:** 10.3389/fpsyt.2021.572445

**Published:** 2021-06-24

**Authors:** Alex Bertrams, Myriam Zäch

**Affiliations:** Educational Psychology Lab, Institute of Educational Science, University of Bern, Bern, Switzerland

**Keywords:** autistic traits, longitudinal study, social anxiety, social contact, university students

## Abstract

Social anxiety (alternatively: *social-contact uncertainty*) in the university context can lead to reduced health, well-being, and performance, and can even cause premature leaving of education. With the present study, we intended to supplement cross-sectional studies on students' autistic traits and social anxiety with longitudinal findings. We measured autistic traits and social-contact uncertainty of 118 university students on two occasions, roughly 1 year apart. Correlation, multiple regression, and cross-lagged analyses showed that more pronounced autistic traits predicted higher future social-contact uncertainty. Social-contact uncertainty did not predict autistic traits. We conclude that university students who are high in autistic traits tend not only to be more socially anxious at the moment but have a heightened risk of still being so in the future.

## Introduction

Due to her political activism, Greta Thunberg's autism diagnosis has come to public awareness. The straightforward appearances of an autistic teenager as a speaker in front of commissions, in numerous interviews, and in the public could give the impression that autistic characteristics result in being unimpressed with challenging social situations. At the theoretical level, it could be argued that more pronounced autistic characteristics are accompanied by lower social motivation and attention (1), potentially leading to emotional indifference with regard to being judged by other people. However, this view is hardly in line with the empirical situation, because evaluative social anxiety is elevated—not attenuated—in autistic people or in people who are high in autistic traits [e.g., (2, 3)]. One can therefore rather assume that the autistic specificities in social cognition, such as difficulties in decoding others' non-verbal behavior [e.g., (4)], lead to poorer interpersonal functioning or social-contact uncertainty even in non-clinical populations (3, 5).

*Social-contact uncertainty* is defined by feelings of personal inadequacy, inferiority, and self-deprecation in social contact; self-doubt, self-insecurity, and negative expectations regarding communication and interpersonal behavior with others are predominant (6) [Note that the official English term *interpersonal sensitivity* may be misleading in the context of autism research; the direct translation of Franke's (6) German term *Unsicherheit im Sozialkontakt* to *social-contact uncertainty* appears to be more useful]. The psychological experiences of high social-contact uncertainty and social anxiety distortion/social phobia, as defined in the Diagnostic and Statistical Manual of Mental Disorders [5th ed.; DSM−5; (7)], overlap to a great extent. In contrast to social anxiety as a disorder, social-contact uncertainty refers to individual differences on a continuum from slight social insecurity to a feeling of total personal deficiency. Gradations in social-contact uncertainty can also be measured and quantified in non-clinical populations such as university students (6).

Freeth et al. (2) pointed out that social anxiety is problematic for students in the UK university context, as the typical work environment is very social; therefore, studying may reduce health, well-being, and performance in socially anxious students and may even cause them to prematurely leave education [see also (8)]. This may also be true for Switzerland, where the present study was conducted; the students frequently have to work together in groups and present their academic works to audiences, and they are required to be in personal contact with their teachers. The reason for socially anxious students' difficulties in such situations may well lie in the adverse self-view, beliefs, and expectations related to social-contact uncertainty as described above.

A few studies have focused on the relationship between autistic traits and social anxiety in university students. These studies found that students higher in autistic traits were more socially anxious [e.g., (2, 9)]. Since these studies were cross-sectional, we aimed at complementing their findings by a longitudinal study on the prediction of social-contact uncertainty (which conceptually overlaps with continuous measures of the psychological experience of social anxiety). We expected that having a higher number of autistic traits would predict higher social-contact uncertainty 1 year later. In contrast, we did not assume that social-contact uncertainty would predict later autistic traits, because this would contradict the notion of autism as a developmental condition [(7); but see (10)].

## Method

### Participants and Procedure

We aimed for a minimum of 110 participants. This size of group would be required to detect even a relationship small in size in a multiple regression model (power analysis with G*Power 3.1: linear multiple regression, random model, a priori; input parameters: two-tailed, H1 ρ2 = 0.10, H0 ρ2 = 0, α = 0.05, 1–β = 0.80, number of predictors = 2). This sample size would also slightly exceed the minimum size of 100 participants for a path model with four variables (11).

The participants were recruited from an email pool of student participants for studies on autistic traits at the Educational Psychology Lab at the University of Bern. The participant pool was originally recruited from regular courses at four universities in the German-speaking part of Switzerland. Only one person in this pool reported having received an autism diagnosis. The university students in this pool had filled out various paper-pencil measures in March/April 2018, including the Autism Spectrum Quotient (AQ) and the subscale Social-Contact Uncertainty from the Brief Symptoms Inventory (BSI). At that time, the present study was not yet planned. In May 2019, the pool members were contacted by email and asked to participate in a short online study. One hundred and twenty students participated; however, two of them stopped the survey and therefore provided incomplete data. Thus, the final sample for the present analyses consists of 118 participants (84/34 female/male; *M*_age_ = 21.81, *SD*_age_ = 3.34). These 118 participants filled out the brief Autism Spectrum Quotient-10 [AQ-10; (12)], the BSI subscale Social-Contact Uncertainty (6), and further measures irrelevant to the present study in May/June 2019. To match their data, the participants provided an anonymous code at both times of measurement (in the following t1/t2 for the first/the second time of measurement).

Only one participant in the final sample indicated having been diagnosed with autism (Asperger syndrome). The mean of the AQ-10 scores at t2 (see Table 1) did not differ from the mean that Allison et al. (12) reported for the AQ-10 in a non-clinical adult sample [*M* = 2.77, *SD* = 2.00, *n* = 419; one sample *t-*test: *t*(117) = −0.73, *p* = 0.47, two-tailed]. This finding suggests that the students who participated were not extraordinarily low or high in autistic traits. The participants were enrolled in a wide range of study programs that included agricultural science (*n* = 7), business studies (*n* = 13), computer science (*n* = 2), economics (*n* = 8), engineering (*n* = 3), environmental science (*n* = 13), food science (*n* = 12), law (*n* = 30), mathematics (*n* = 2), psychology (*n* = 1), social science (*n* = 9), and teacher training (*n* = 18). The average number of semesters of studying these programs was 3.03 (*SD* = 1.71) at t1. No further in-depth socioeconomic data were assessed, due to the protection of privacy (information about ethnicity could allow conclusions about individual persons in this university sample) and time constraints.

**Table 1:** Descriptive statistics and correlations of the applied psychometric measures.

						**Correlations**		
**Measure**	**ω**	***M***	***SD***	**Possible range**	**Observed range**	**1**	**2**	**3**
1. Autistic traits at t1	0.89	8.47	5.12	0–32	1–24	–		
2. Autistic traits at t2	0.72	2.64	2.00	0–10	0–9	0.64	–	
3. Social-contact uncertainty at t1	0.84	9.50	4.15	4–20	4–20	0.51	0.32	–
4. Social-contact uncertainty at t2	0.84	9.44	3.88	4–20	4–20	0.48	0.39	0.58

*N = 118. Overall scores for autistic traits and social-contact uncertainty were obtained by summing up the responses to the scale items. The measures for autistic traits at t1 and t2 differed in their number of items, which affects their possible ranges. Coding for biological sex: 1 = female, 2 = male. All ps for correlations were < 0.001 (two-tailed)*.

### Psychometric Measures

#### Autistic Traits

At both times of measurement, the AQ was applied—the brief version AQ-k at t1 (13) and the brief version AQ-10 at t2 (12). The AQ (and the brief versions used) consists of items tapping different diagnostic features of autism (e.g., “When I'm reading a story, I find it difficult to work out the characters' intentions”) that are rated on a four-point scale from definitely agree to definitely disagree. Definitely/slightly agree and definitely/slightly disagree are awarded one and zero points, respectively (or zero/one points when the items are reversed), and all points are summed to form a total score. Higher total scores reflect a higher number of autistic traits.

#### Social-Contact Uncertainty

The BSI has been widely used in university student samples and is well-suited for the assessment of mental stress in such samples (6). The subscale *Social-Contact Uncertainty* from the BSI (6) was used to measure social-contact uncertainty at t1 and t2. The four items refer to experiences during the previous seven days (e.g., “How much you have suffered over the past seven days from the feeling that people are unfriendly or do not like you”). The answers are given on a five-point scale from not at all to very much. According to the participant's rating of an item, a point from 1 to 5 is awarded. The points for all four items are summed up to a total score. Higher total scores reflect higher social-contact uncertainty.

### Analysis Strategy

To analyze the relationship between autistic traits and social-contact uncertainty, we first calculated Pearson correlation coefficients. Next, we conducted a hierarchical multiple regression analysis to predict social-contact uncertainty at t2 by autistic traits at t1 while holding constant the baseline social-contact uncertainty at t1. In this article, we report a repeated version of this regression analysis in which age and biological sex were also statistically controlled. Finally, to take into account all interrelations, we performed path analysis in AMOS version 20 (14) with maximum likelihood estimation. The cross-lagged approach was applied [e.g., (10)]. Because the cross-lagged model was saturated (i.e., *df* = 0), it did not allow interpreting fit statistics; however, it was still possible to generate and evaluate the parameter estimates (15). In all analyses, we sought evidence that autistic traits are predictive of social-contact uncertainty over time.

## Results

Age at t1 was not correlated with autistic traits or social-contact uncertainty at t1 or t2 (|*r*|s < 0.12, *p*s > 0.21). In addition, a row of independent samples *t*-tests indicated no significant differences between female and male participants in autistic traits or social-contact uncertainty at t1 or t2 (*t*s <1.63, *p*s > 0.10). Thus, age and biological sex were not related to the main variables and had no role in the relational pattern under investigation.

**Figure 1:**
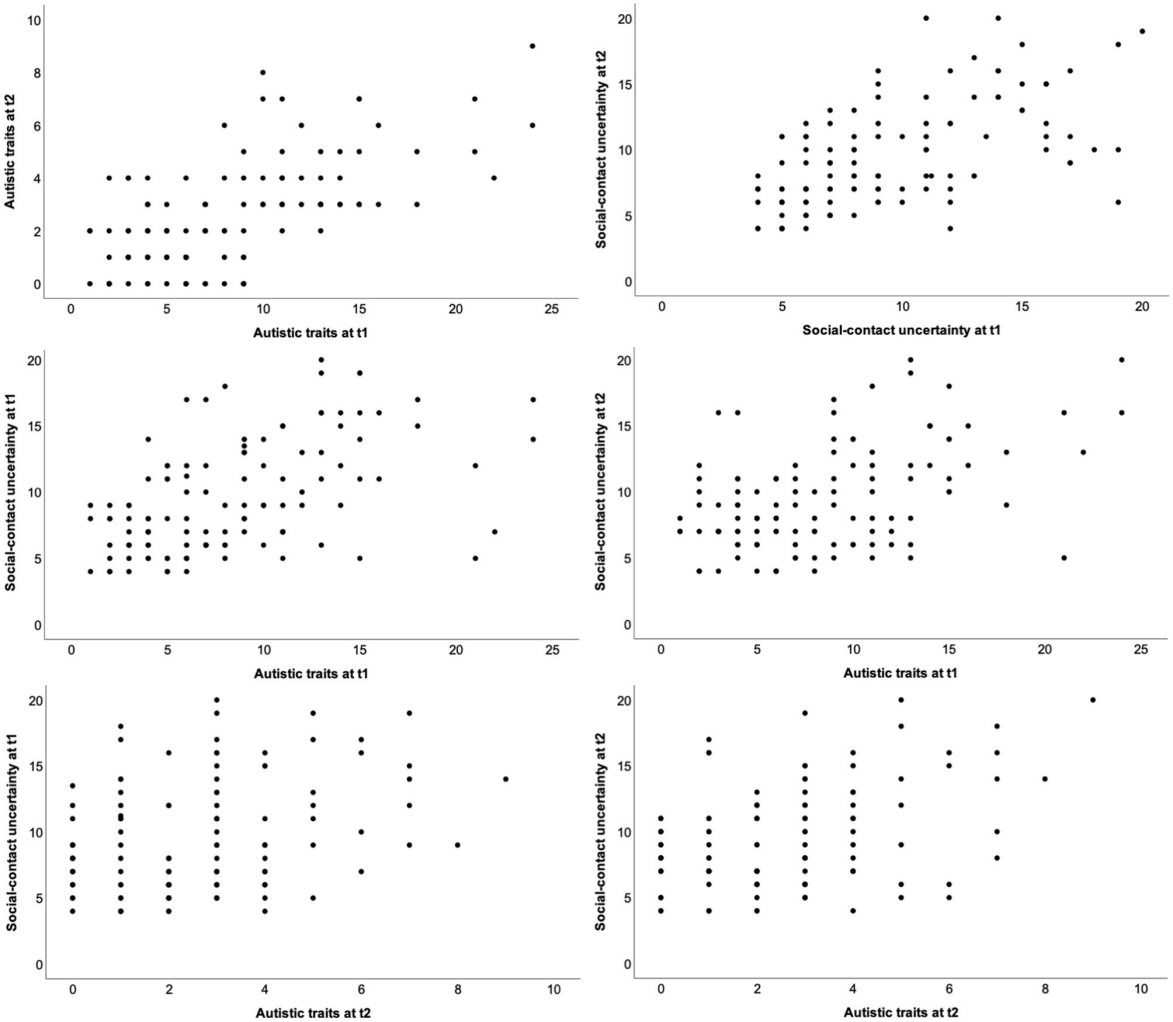
Scatterplots for the intercorrelations of autistic traits and social-contact uncertainty at both times of measurement.

Table 1 shows the descriptive statistics and intercorrelations of autistic traits and social-contact uncertainty at both times of measurement. As can be seen there, these variables were positively correlated with each other; that is, autistic traits and social-contact uncertainty were cross-sectionally as well as longitudinally related. Figure 1 depicts the scatterplots for all intercorrelations.

Table 2 presents the results of the hierarchical multiple regression. The analysis revealed that social-contact uncertainty at t1 predicted social-contact uncertainty at t2 over and above autistic traits at t1. Most importantly, autistic traits at t1 predicted social-contact uncertainty at t2 over and above social-contact uncertainty at t1. The results were independent of age and biological sex, which were not significant predictors of social-contact uncertainty at t2. The results were the same when age and biological sex were not included in the model.

**Table 2:** Hierarchical multiple regression analysis regressing social-contact uncertainty at t2.

**Blocks and predictors**	***B***	***SE B***	**β**	***t***	***p***	***R*^**2**^**	**Δ*R*^**2**^**	***p* for Δ*R*^**2**^**
Block 1						0.01	0.01	0.49
Age at t1	−0.07	0.11	–0.06	–0.60	0.55			
Biological sex	–0.72	0.81	–0.08	–0.89	0.38			
Block 2						0.34	0.33	<0.001
Age at t1	–0.01	0.09	–0.01	–0.08	0.94			
Biological sex	–0.08	0.67	–0.01	–0.11	0.91			
Social-contact uncertainty at t1	0.54	0.07	0.58	7.50	<0.001			
Block 3						0.39	0.05	0.004
Age at t1	–0.01	0.09	–0.004	–0.05	0.96			
Biological sex	–0.53	0.67	–0.06	–0.79	0.43			
Social-contact uncertainty at t1	0.41	0.08	0.44	4.96	<0.001			
Autistic traits at t1	0.20	0.07	0.26	2.91	0.004			

*N = 118. Coding for biological sex: 1 = female, 2 = male*.

**Figure 2:**
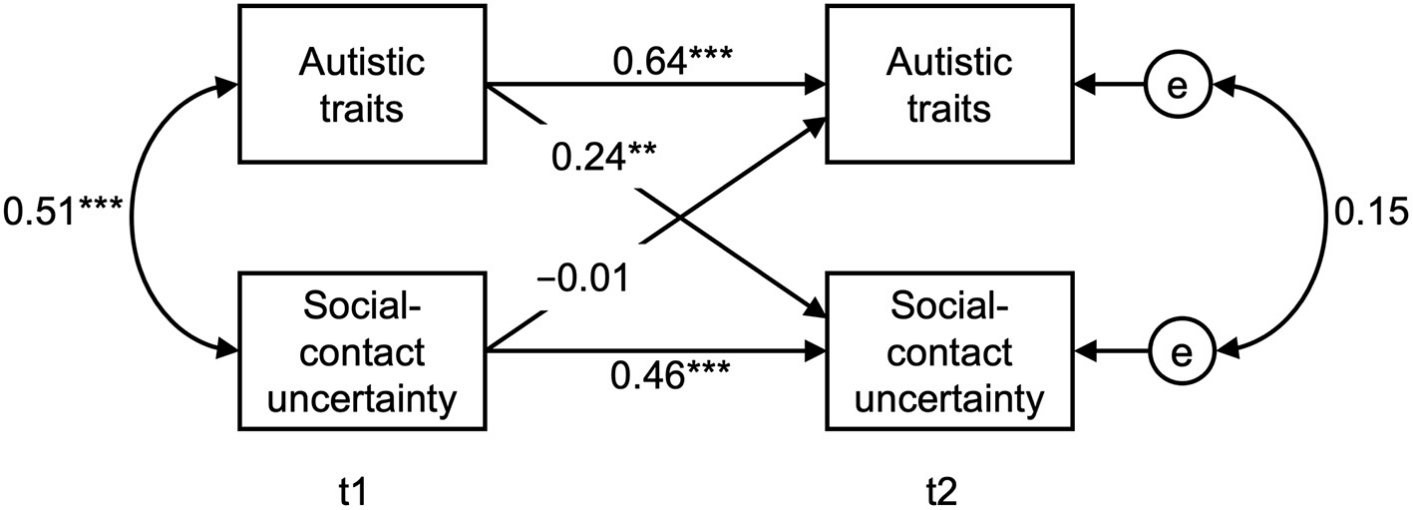
Cross-lagged model to predict autistic traits and social-contact uncertainty over time. Correlations and standardized regression weights are shown. *N* = 118. *p < 0.05, **p < 0.01, ***p < 0.001 (all two-tailed).

The cross-lagged model and the parameters are depicted in Figure 2. As can be seen there, over and above the stability parameters for autistic traits and social-contact uncertainty, autistic traits predicted social-contact uncertainty over time. In contrast, social-contact uncertainty did not predict future autistic traits (*p* = 0.95).

## Discussion

In the present study, we found that university students' autistic traits predicted their social-contact uncertainty. The higher their autistic traits, the higher was the students' social-contact uncertainty (slightly more than) 1 year later—in bivariate correlation, multiple regression, and cross-lagged analyses. This finding complements the few existing studies with non-clinical university student samples that showed a cross-sectional relationship between autistic traits and social anxiety (2, 9).

According to Cage and Howes (16), appropriate mental health support for autistic people is needed within university. Our results suggest that students high in autistic traits tend not only to be more socially anxious at the moment but also have a heightened risk of still being so in the future. One implication is that students with a high number of autistic traits are prone to enduring stress, experiencing reduced performance, and even prematurely leaving education, just because studying at university requires frequent social contact and interaction (2). Therefore, treatment for social anxiety for autistic people [(e.g., (17)] and systematic support at universities (16) could be useful for people with many autistic traits, regardless of a formal autism diagnosis. Notably, only a portion of the people who are high in autistic traits and seek a diagnostic assessment of autism eventually qualify for an autism diagnosis (18). Moreover, autistic traits may be present in neurodevelopmental conditions other than autism [e.g., (19)]. The present results may be relevant to these individuals as well.

Future research may address the limitations of the present study. For example, future studies might replicate the findings with a larger sample size, and a more balanced gender distribution could preclude the possibility of bias due to the female overrepresentation in the present study. The addition of consecutive time points of measurement could also be helpful to expand the findings on the relationship between autistic traits and social-contact uncertainty by allowing causal conclusions (20). Other limitations derive from the brevity of our follow-up survey, which was intended to encourage willingness to participate. For example, we did not measure and control for the variety of clinical conditions that might influence the autistic traits–social-contact uncertainty relationship.

The results of the cross-lagged model must also be interpreted with caution. Since the version of the AQ that was applied for the follow-up measurement was shorter than the version for the first measurement, it is possible that the two versions measured differently. Measuring autistic traits with the AQ-10 at t2 may also constitute a limitation since this measure was originally reserved for screening in primary care settings as a guide for referrals (12). However, the limitations concerning the use of the AQ-10 do not affect the hierarchical multiple regression analysis predicting social-contact uncertainty.

Finally, further longitudinal studies could assess additional variables that may be relevant in the autistic traits–social-contact uncertainty relationship. Such variables may refer, for example, to the potential consequences of elevated social-contact uncertainty. In this regard, because of social-contact uncertainty, students who are high in autistic traits might be prone to experiencing enduring discomfort and distress in social university settings (2). In addition, future research could explore whether the autistic traits of students are related to the consumption of drugs or engagement in other addictive behaviors as strategies for coping with social-contact uncertainty (21).

## Data Availability Statement

The raw data supporting the conclusions of this article will be made available by the authors, without undue reservation.

## Ethics Statement

The studies involving human participants were reviewed and approved by ethics committee of the Faculty of Human Sciences at the University of Bern. The patients/participants provided their written informed consent to participate in this study.

## Author Contributions

AB conceived the project, designed the study, conducted the study, analyzed the data, and wrote the manuscript. MZ conducted the study, prepared the data set, and wrote the manuscript. All authors contributed to the article and approved the submitted version.

## Conflict of Interest

The authors declare that the research was conducted in the absence of any commercial or financial relationships that could be construed as a potential conflict of interest.
